# Emodin Protects against Diabetic Cardiomyopathy by Regulating the AKT/GSK-3β Signaling Pathway in the Rat Model

**DOI:** 10.3390/molecules190914782

**Published:** 2014-09-17

**Authors:** Zhiqin Wu, Qingwei Chen, Dazhi Ke, Guiqiong Li, Wei Deng

**Affiliations:** Geriatrics, the 2nd Affiliated Hospital of Chongqing Medical University, Chongqing 400010, China; E-Mails: wuzqchina@gmail.com (Z.W.); kedazhili@163.com (D.K.); lgqwg@aliyun.com (G.L.); dengwei1176@163.com (W.D.)

**Keywords:** emodin, diabetic cardiomyopathy, blood glucose, Akt, GSK-3β

## Abstract

Diabetes mellitus (DM) has been recognized as a major health problem. Emodin (Emo) has been reported to exhibit protective effects against diabetic nephropathy. However, little has been known about the effect of Emo on diabetic cardiomyopathy (DCM). A type 2 DM model was induced in rats by low dose streptozotocin (STZ) combined with high energy intake. We found that Emo-treated groups displayed significantly higher body weight (BW) and lower heart weight (HW)/BW. Furthermore, Emo could significantly decrease blood glucose, total cholesterol (TG) levels, and triglyceride (TC) levels in diabetic rats. Moreover, the Emo-treated group showed a marked increase in heart rate (HR) and showed lower left ventricular end-diastolic diameter (LVEDD), left ventricular end-systolic diameter (LVESD), left ventricular posterior wall thickness (LWPWT), and interventricular septal diastolic wall thickness (IVSD). Emo induced a significant increase in phosphorylation of Akt and GSK-3β in myocardium. These results suggest that Emo may have great therapeutic potential in the treatment of DCM by Akt/GSK-3β signaling pathway.

## 1. Introduction

Diabetes mellitus (DM) is a group of metabolic diseases characterized by high levels of blood sugar, which are due to problems in insulin secretion or insulin, or both. DM can lead to many complications, including diabetic ketoacidosis and nonketotic hyperosmolar coma, as well as heart disease, stroke, kidney failure, foot ulcers, and damage to the eyes [1,2]. Currently, DM has been recognized as a major health problem [3], because the number of diabetic patients is increasing rapidly and is expected to reach 439 million worldwide by 2030 [4]. At present, type 2 diabetes (T2D) accounts for 90%–95% of all diagnosed diabetes in adults [5]. The leading cause of death in diabetic patients is cardiovascular disease, including heart failure. The underlying causes of diabetes-associated heart disease are multifactorial. DCM is characterized by myocardial dysfunction in the absence of coronary artery disease, hypertension, or valvular heart disease as an independent diabetic cardiac complication [6,7,8].

DCM is a disorder of the heart muscle in people with diabetes, and is associated with both type 1 and type 2 DM. DCM can cause diastolic and systolic dysfunction damage, resulting in myocardial ischemia and heart failure. At present, DCM is a significant contributor to the morbidity and mortality associated with diabetes and metabolic syndrome [7]. Myocardial lip metabolism, inflammation, oxidative stress, myocardial fibrosis, myocardial apoptosis and mitochondrial damage are considered possible mechanisms for the development and progression of DCM [9,10,11,12,13]. However, the development of DCM has been poorly understood, and the underlying mechanisms have not been completely elucidated.

Emodion (6-methyl-1,3,8-trihydroxyanthraquinone. Emo) is a purgative resin obtained from *Rheum emodi* (a Himalayan rhubarb). It was known that Emo has been used extensively in traditional Chinese medicine, as it is nontoxic and has a variety of therapeutic properties, including antioxidant properties, anti-inflammatory properties, anti-virus properties, and so on [14,15,16,17,18,19]. In the past two decades, Emo has also been found to reverse insulin resistance, hyperglycemia, and other symptoms linked to obesity and obesity-related metabolic diseases [20,21,22,23]. Emo has recently been reported to exhibit protective effects against endothelial cell dysfunction by increasing the release of nitric oxide (NO) and by inhibiting the resctive oxygen species (ROS) production [24,25]. However, little has been known about the effect of Emo on DCM.

Since Emo has antioxidant, anti-inflammatory and lipid-lowering effects, and oxidative stress and inflammation are important factors to induce the formation of DCM, we therefore speculated that Emo might possesses protective effects against DCM. In this study, a rat model of type 2 DM was induced by high fat chow and low dose STZ injection, with the intention of investigating the potential effects of Emo in a rat model of type 2 DM, as well as the possible mechanism of Emo’s effects.

## 2. Results and Discussion

### 2.1. Effect of Emo on BW in STZ-Induced Diabetic Cardiomyopathy

**Figure 1:**
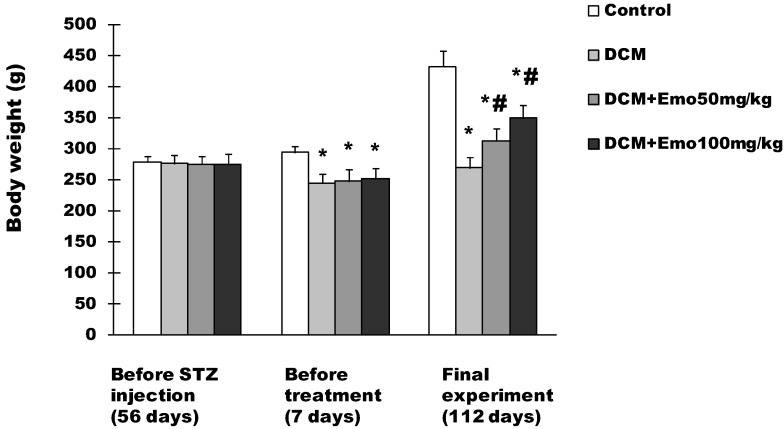
Effect of emodin on body weight (BW) in STZ-induced diabetic cardiomyopathy. Data are means ± SEM; * *p* < 0.05 *vs*. control group; ^#^ *p* < 0.05 *vs*. DCM group; n = 10 per group.

Before STZ injection, the BW of the rats was 279 ± 9 g, 276 ± 14 g, 275 ± 13 g, and 274 ± 17 g, respectively in control, DCM, DCM + 50 mg/kg Emo, and DCM + 100 mg/kg Emo group, and there was no difference in BW of rats in each group (Figure 1). One week after STZ injection, BW of the rats was 294 ± 11 g, 245 ± 14 g, 248 ± 18 g, and 251 ± 17 g, respectively in the control, DCM, DCM + 50 mg/kg Emo, and DCM + 100 mg/kg Emo group (Figure 1). Compared with the control group, the DCM, DCM + 50 mg/kg Emo, and DCM + 100 mg/kg rats had markedly lower BW (*p* < 0.05); there are no differences in the DCM, DCM + 50 mg/kg Emo, and DCM + 100 mg/kg groups (Figure 1). Compared with the DCM group, 50 mg/kg Emo-treated and 100 mg/kg Emo-treated groups gained significantly higher BW (*p* < 0.05) (Figure 1). These results indicate that Emo can increase animal BW.

### 2.2. Effect of Emo on Blood Glucose in STZ-Induced Diabetic Cardiomyopathy

**Figure 2:**
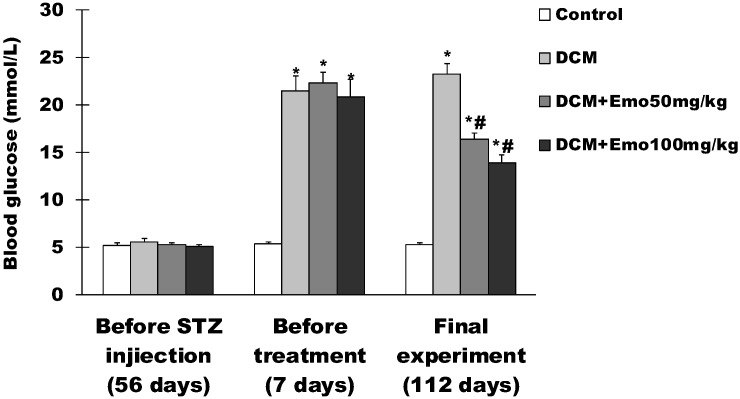
Effect of emodin on blood glucose in STZ-induced diabetic cardiomyopathy. Data are means ± SEM; * *p* < 0.05 *vs.* control group; ^#^ *p* < 0.05 *vs.* DCM group; n = 10 per group.

As shown in Figure 2, before STZ-induced diabetic cardiomyopathy, fasting blood glucose levels were 5.2 ± 0.3 mmol/L, 5.6 ± 0.3 mmol/L, 5.3 ± 0.2 mmol/L, and 5.1 ± 0.2 mmol/L, respectively in control, DCM, DCM + 50 mg/kg Emo, and DCM + 100 mg/kg Emo group, and there was no difference in blood glucose of rats in each group. One week after STZ-induced diabetic cardiomyopathy, the fasting blood glucose levels were 5.4 ± 0.2 mmol/L, 21.5 ± 1.6 mmol/L, 22.3 ± 1.2 mmol/L, and 20.9 ± 1.8 mmol/L respectively in control, DCM, DCM + 50 mg/kg Emo, and DCM + 100 mg/kg Emo group (Figure 2). Compared with the control group, the fasting blood glucose levels increased significantly in the DCM, DCM + 50 mg/kg Emo, and DCM + 100 mg/kg Emo groups (*p* < 0.05). At the end of the experiment, the fasting blood glucose levels were 16.4 ± 0.9 mmol/L and 13.9 ± 0.7 mmol/L, respectively in the DCM + 50 mg/kg and DCM + 100 mg/kg Emo groups (Figure 2). Compared with the DCM group, Emo-treated rats displayed significantly decreased blood glucose levels, suggesting that Emo can significantly decrease blood glucose levels in diabetic rats.

### 2.3. Effects of Emo on HW and HW/BW STZ-Induced Diabetic Cardiomyopathy

**Figure 3:**
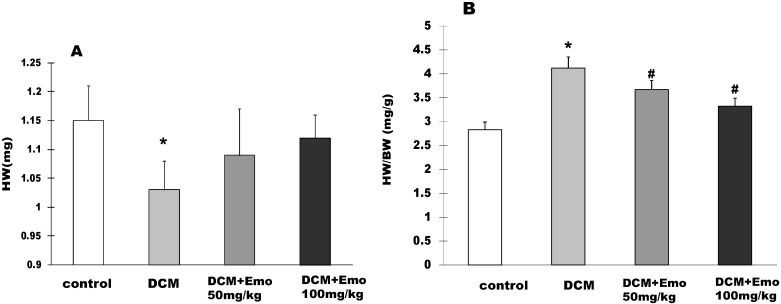
Effects of emodin on HW and HW/BW STZ-induced diabetic cardiomyopathy. (**A**) HW; (**B**) HW/BW. Data are means ± SEM; * *p* < 0.05 *vs.* control group; ^#^ *p* < 0.05 *vs.* DCM group; n = 10 per group.

At the end of the experiment, the HW of the rats was 1.15 ± 0.06 g, 1.03 ± 0.05 g, 1.09 ± 0.08 g, and 1.12 ± 0.04 g, respectively in control, DCM, DCM + 50 mg/kg Emo, and DCM + 100 mg/kg Emo group (Figure 3A). Compared with the control group, the DCM group rats had a significant decrease in HW, but Emo-treated rats had no difference (Figure 3A). In order to better show the case of cardiac hypertrophy, HW/BW was measured in STZ-induced diabetic cardiomyopathy. As shown in Figure 3B, HW/BW was 2.83 ± 0.16 in the control group, while HW/BW was 4.12 ± 0.23 in the DCM group. It was significantly higher than the control group, suggesting that cardiac hypertrophy was present in the DCM group rats. On the other hand the Emo-treated group had significantly lower HW/BW (*p* < 0.05) (Figure 3B).

### 2.4. Emo Alleviated Left Ventricular Dysfunction

As shown in Table 1, the DCM group had a slower heart rate (HR) than the control group, while HR was significantly increased in the Emo-treated groups compared to the DCM group (*p* < 0.05). The following had higher in the DCM group than the control group did (*p* < 0.05): the LVEDD, LVESD, LWPWT, and IVSD. Left ventricular fractional shortening (FS) and ejection fraction (EF) had lower values in the DCM group than the control group did (*p* < 0.05) (Table 1). On the contrary, the Emo-treated groups showed lower left ventricular inner diameter, LWPWT, and IVSD than that of the DCM group did (*p* < 0.05) (Table 1). These results indicated that Emo could inhibit interventricular septal hypertrophy and increased the left ventricular FS and EF.

**Table 1:** Emodin alleviated diabetic cardiomyopathy induced left ventricular dysfunction. Data are means ± SEM; * *p* < 0.05 *vs.* control group; ^#^*p* < 0.05 *vs.* DCM group; n = 5 per group.

Group	HR (bpm)	LWPWT (mm)	IVSD (mm)	LVEDD (mm)	LVESD (mm)	FS (%)	EF (%)
**Control**	384 ± 15	1.49 ± 0.09	1.34 ± 0.07	2.83 ± 0.21	1.58 ± 0.11	44.2 ± 2.7	82.6 ± 2.4
**DCM**	311 ± 11 *	1.74 ± 0.11 *	1.81 ± 0.14 *	3.48 ± 0.31 *	2.31 ± 0.28 *	33.6 ± 3.2 *	70.8 ± 3.4 *
**DCM+Emo 50 mg/kg**	332 ± 13 ^#^	1.56 ± 0.13	1.55 ± 0.09 ^#^	3.11 ± 0.16	1.84 ± 0.19 ^#^	40.8 ± 2.9	79.3 ± 2.8
**DCM+Emo100 mg/kg**	358 ± 14 ^#^	1.63 ± 0.15 ^#^	1.49 ± 0.11 ^#^	2.89 ± 0.23 ^#^	1.69 ± 0.16 ^#^	41.5 ± 2.8 ^#^	80 ± 2.6 ^#^

### 2.5. Effect of Emo on TG and TC in STZ-Induced Diabetic Cardiomyopathy

**Figure 4:**
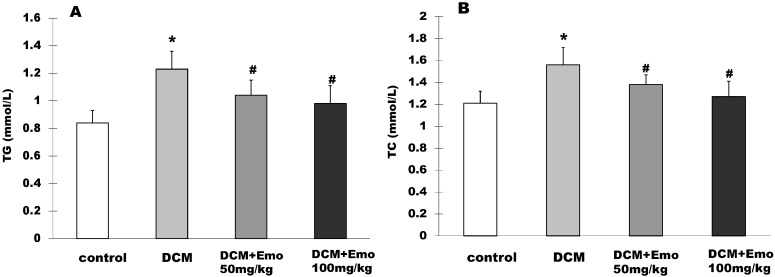
Effect of emodin on TG and TC in STZ-induced diabetic cardiomyopathy. (**A**) TG; (**B**) TC. Data are means ± SEM; * *p* < 0.05 *vs.* control group; ^#^ *p* < 0.05 *vs.* DCM group; n = 5 per group.

The rat serum TG level was 0.84 ± 0.09 mmol/L in the control group, while in the DCM group it was 1.23 ± 0.13 mmol/L and it showed significantly higher TG in the basal fasting state (*p* < 0.05) (Figure 4A). The 50 mg/kg and 100 mg/kg Emo groups were 1.04 ± 0.11 mmol/L and 0.98 ± 0.13 mmol/L in the TG level, respectively, and showed significantly lower TG than the DCM group (*p* < 0.05) (Figure 4A). As shown in Figure 4B, the rat serum TC level was 1.56 ± 0.16 mmol/L in the DCM group, the control group was 1.21 ± 0.11 mmol/L and showed significantly higher TC in the basal fasting state (*p* < 0.05) (Figure 4B). The 50 mg/kg and 100 mg/kg Emo groups were 1.38 ± 0.09 mmol/L and 1.27 ± 0.14 mmol/L in the TC level, respectively, and showed significantly lower TG than the DCM group (*p* < 0.05) (Figure 4B).

### 2.6. Effect of Emo on AKT/GSK-3β Signaling Pathway in STZ-Induced Diabetic Cardiomyopathy

**Figure 5:**
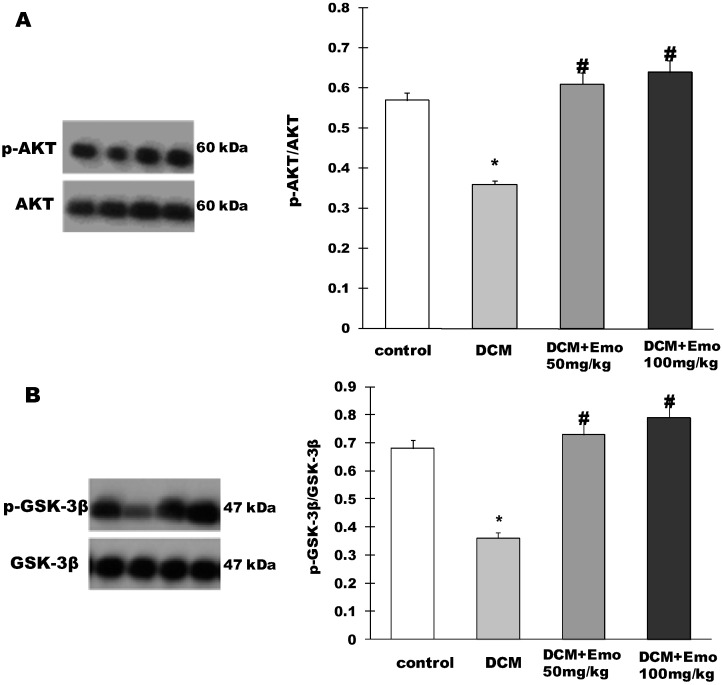
Emodin inactivated AKT and GSK-3β of heart in STZ-induced diabetic cardiomyopathy. (**A**) p-AKT/AKT; (**B**) p-GSK-3β/GSK-3β. Data are means ± SEM; * *p* < 0.05 *vs.* control group; ^#^ *p* < 0.05 *vs.* DCM group; n =5 per group.

As shown in Figure 5A, AKT phosphorylation was significantly blocked in the DCM group compared to the control group. However, 50 and 100 mg/kg Emo groups showed a significant increase in phosphorylation of Akt (Figure 5A). In addition, GSK-3β phosphorylation was significantly hampered in the DCM group compared to the control group. On the contrary, 50 or 100 mg/kg Emo induced a significant increase in phosphorylation of GSK-3β (*p* < 0.05) (Figure 5A).

### 2.7. Disscussion

DCM was first recognized by Rubler *et al*. in diabetics with congestive heart failure (CHF), but they had no evidence of coronary atherosclerosis [26]. DCM is different from coronary artery disease or hypertension and is a specific cardiomyopathy caused by diabetes. DCM can cause left ventricular hypertrophy, left ventricular diastolic dysfunction, and systolic dysfunction. Many studies suggest that DCM is caused by multiple factors, such as high blood sugar, cholesterol, and hyperinsulinemia, which can induce changes in the downstream transcription factors, leading to myocardial cell growth and proliferation and endothelial function changes [27,28,29,30]. However, the pathogenesis of DCM has not been fully elucidated.

In order to understand the pathogenesis of DCM, animal models have been used [31,32,33]. However, in some animal models of DCM, drug treatment, or the effects of genetic mutation often lead to obesity and diabetes. All of these models have limitations and none are a perfect phenocopy of the human condition [34]. Currently, there are animal models of type 2 diabetes induced by chemical agents [35,36]. In this study, we selected Wistar rats (160–180 g), which were fed a high energy diet for 8 weeks, after which the rats were injected with STZ (50 mg/kg). The rat model of type 2 diabetes was successfully established by the diagnostic criteria of diabetic rats.

The study has shown that Emo can reduce fasting blood glucose, resulting in inhibition of lipolysis in diabetic rats [20,22]. In this study, diabetic rats that were treated with Emo showed significantly decreased blood glucose levels and gained significantly higher BW (*p* < 0.05) (Figure 1 and Figure 2). DCM rats showed that serum TG and TC levels were increased significantly compared to control rats. This kind of increase is associated with type 2 diabetes, which has similar clinical symptoms. Diabetic rats treated with Emo significantly decreased serum TG and TC levels (*p* < 0.05) (Figure 4).

The study found that diabetic patients without other heart-related diseases for more than five years showed enlarged left atria and left ventricles, and the disease time to grow more, left atrium and left ventricle enlargement and more obvious [37]. Using echocardiography at the end of the experiment, we found that LVEDD, LVESD, LWPWT, and IVSD were higher in the DCM group than in the control group (*p* < 0.05) (Table 1). However, the DCM rats that were treated with Emo had significantly reduced left ventricular diameter, LWPWT, and IVSD thinning. Atrial and ventricular chamber enlargements are some of the most important pieces of evidence that indicate structural damage and decreased cardiac function of the important reasons by DCM. Patel *et al.* found that there were 73% of patients with abnormal echocardiogram among 495 diabetic patients, including 8% of patients with left ventricular hypertrophy, 74% of patients with diastolic dysfunction, and 18% of patients with diastolic and systolic dysfunction [38]. Ventricular FS and EF are the indicators used most commonly in clinical evaluation of the cardiac systolic function. FS and EF were significantly higher in the Emo-treated groups than in the DCM group (*p* < 0.05) (Table 1), indicating that Emo can improve diabetes-induced systolic dysfunction.

The metabolic syndrome is a clustering of components known to promote the development of DM and heart disease. The previous studies have shown that Emo could protect rats, which was treated with high fat/high fructose diet, from insulin resistance, hypertriglyceridaemia and systemic necro-inflammation [15]. In this study, our results demonstrated that Emo could significantly decrease blood glucose, total cholesterol (TG) levels, and triglyceride (TC) levels in diabetic rats. This highlights the potential value of Emo for the treatment of metabolic syndrome or type 2 diabetes. Emo plays an anti-inflammatory and anti-cancer role in several inflammatory or cancer diseases, because Emo is a pleiotrophic molecule capable of interacting with several major molecular targets [39]. In addition, Emo isolated from *Rheum emodi* should be relatively nontoxic and do not cause side effects. However, some studies have reported that Emo has NTP toxicology and carcinogenicity [40]. This is one of the important issues worthy of attention in animal studies of Emo.

Type 2 diabetes, diabetic nephropathy and cardiovascular disease are closely related to impaired AKT/GSK-3β pathways [41,42,43]. Phosphorylated AKT can reduce free fatty acids and inflammatory cytokines in diabetes. In addition, AKT can regulate cardiovascular functions, including coronary angiogenesis, the growth of myocardial cells and cardiac systolic function [42]. GSK-3β plays an important role in glycogen metabolism and insulin resistance [41,44]. In this study, we found that Emo can induce a significant increase in phosphorylation of Akt and GSK-3β, indicating that AKT/GSK-3β plays an important role in the prevention of DCM.

## 3. Experimental Section

### 3.1. Reagents and Antibodies

STZ and Emo were purchased from Sigma-Aldrich (St. Louis, MO, USA); the HF diet (34.5% fat, 17.5% protein, 48% carbohydrate) was obtained from Beijing HFK Bio-Technology (Beijing, China); The anti-AKT, anti-GSK3β, and phosphorylated-specific anti-AKT (Ser473), anti-GSK3β (Ser9) antibodies were purchased from Cell Signaling Technology (Danvers, MA, USA).

### 3.2. Rat Model of Type 2 Diabetes 

Sixty male Wistar rats (160–180 g) were purchased from the Animal Research Centre of the Third Military Medical University (Chongqing, China). The animals were housed in standard cages and maintained under controlled room temperature and humidity with 12/12-hour light-dark cycles. After 1 week of acclimatization, the rats were then randomized into two groups: control (n = 10) and diabetes (n = 50). Diabetic group rats were fed an HF diet, and the control rats received normal chow. Eight weeks later, the diabetic group of rats was induced by a single intraperitoneal injection of STZ (50 mg/kg in 0.1 mol/L citrate buffers, pH 4.5). The control group received citrate buffer (intraperitoneally) alone. Thus, the zero time point is 56 days before the injection of STZ. One week after STZ administration, rats with fasting blood glucose > 11.1 mmol/L in two consecutive analyses were considered the diabetic rat model ones. The time point is 7 days before treatment. The experiments were performed according to national regulations and approved by the local animal ethics committee.

### 3.3. Experimental Design

Diabetic rats were then randomized into three groups: DCM, DCM + 50 mg/kg Emo and DCM + 100 mg/kg Emo. There are 10 rats in each group. DCM + 50 mg/kg and DCM + 100 mg/kg groups were treated daily with 50 mg/kg and 100 mg/kg of Emo by the route of ig, respectively. Control and DCM groups were treated daily with carboxymethylcellulose sodium (ig) alone. The animals were treated with Emo for 16 weeks; animals were placed under light anesthesia with pentobarbital sodium. The time point for the final experiment is 112 days. Body weights were recorded weekly.

### 3.4. Blood Analyses

After the rats had fasted overnight, blood samples were collected by tail vein blood. Total cholesterol, triglyceride levels, and FBG were analyzed with use of the Bayer 1650 Blood Chemistry Analyzer (Bayer, Tarrytown, NY, USA).

### 3.5. Heart Weight and Body Weight

At the end of the experiment, HW and BW were measured and the ratios of HW and BW were calculated.

### 3.6. Echocardiography

The rats were anesthetized with 1% sodium pentobarbital and were fixed on the board. The hairs of rats were removed in the ventral chest area and front area. There is the echocardiography probe which emits and receives ultrasound in the range of 5–12 MHz. The LVEDD, LVESD, LWPWT, and IVSD were measured. The FS and EF were calculated using the formula as follows:

FS = [(LVEDD − LVESD)/LVEDD] × 100%
(1) [45]

EF = [(LVEDD^3^ − LVEDS^3^)/LVEDD^3^] × 100% (2) [46]

### 3.7. Western Blotting

Frozen left ventricular tissue was weighed and diced into small pieces in RIPA buffer (150 mM NaCl, 1% sodium deoxycholate, 1% Triton X-100, 0.1% SDS, 10 mM Tris-HCl pH 7.2), separated by SDS-PAGE, and transferred onto nitrocellulose membranes (Millipore, Boston, MA, USA). Primary antibodies against the following proteins were used: AKT, GSK-3β, P-AKT, and P-GSK-3β. Primary antibodies were detected by using appropriate horseradish peroxidase-conjugated secondary antibodis. Blots were developed by the chemiluminescent detection system from Pierce (Rockford, IL, USA). To demonstrate equal loading, blots were stripped and reprobed with a specific antibody recognizing β-actin (Sigma-Aldrich), which was used to assess the phosphor/total proteins ratio.

### 3.8. Statistical Analysis

Statistical significance was assessed with a two-tailed Student’s t-test between two groups and by one-way ANOVA for comparisons among multiple groups. Statistical significance was defined as *p* < 0.05. Statistical analyses were performed with Statistical Product and Service Solutions (SPSS) Windows 13.0 Statistical Software (SPSS Inc., Chicago, IL, USA).

## 4. Conclusions

In conclusion, our results demonstrate that Emo provides potent protection against diabetic cardiomyopathy in a rat model by regulating the AKT/GSK-3β signaling pathway. Our discovery suggests that Emo has great potential as an anti-diabetic cardiomyopathy therapeutic agent.
